# Incidence of Non-Melanoma Skin Cancers in Salento (Southern Italy): A 15-Year Retrospective Analysis from the Cancer Registry of Lecce

**DOI:** 10.3390/epidemiologia5010001

**Published:** 2023-12-21

**Authors:** Emiliano Sordi, Prisco Piscitelli, Carla Albanese, Anna Melcarne, Anna Tardio, Fabrizio Quarta, Enrico Greco, Alessandro Miani, Andrea Falco, Elisabetta De Matteis, Maurizio Congedo, Adele Civino

**Affiliations:** 1 Local Health Authority ASL Le, 73100 Lecce, Italy; emilianosordi72@gmail.com (E.S.); melcarneanna@gmail.com (A.M.); annatardio@hotmail.it (A.T.); uose@asl.lecce.it (F.Q.); dr.dematteis.elisabetta@gmail.com (E.D.M.); mauriziocongedo@gmail.com (M.C.); adelecivino@gmail.com (A.C.); 2 Department of Experimental Medicine, University of Salento, 73100 Lecce, Italy; 3 Experimental Zooprophylactic Institute of Apulia and Basilicata (IZSPB), 71121 Foggia, Italy; carla.albanese8@gmail.com; 4 Department of Chemical and Pharmaceutical Sciences, University of Trieste, 34100 Trieste, Italy; enrico.greco@units.it; 5 Italian Society of Environmental Medicine (SIMA), 20123 Milan, Italy; alessandro.miani@gmail.com; 6 Department of Health Sciences, European University of Madrid, 28670 Madrid, Spain; falco.and@gmail.com

**Keywords:** non-melanoma skin cancers, basal-cell carcinoma, squamous-cell carcinoma, incidence, epidemiology

## Abstract

Background and Objectives: Non-melanoma skin cancers (NMSCs) include basal cell carcinoma (BCC) and squamous-cell carcinoma (SCC), as well as a wide range of rare skin tumors. NMSCs is the most frequently diagnosed type of tumor among Caucasians. We aimed at estimating the incidence and mortality of NMSCs in the Salento area (Lecce province, Southern Italy), whose population is assumed to experience heavy and frequent sun exposure due to climatic/environmental factors, both for working and leisure activities. Materials and Methods: We computed the incidence of NMSCs in the Province of Lecce by examining the comprehensive real-world data collected by the local cancer registry, which covers all the 830,000 inhabitants, over a period of fifteen years (from 2003 to 2017), with a focus on the latest 5 years (2013–2017) for the analysis of the different histologic morphologies of these tumors. The incidence of NMSCs has been described in terms of absolute frequencies, crude rates and age-adjusted direct standardized rates (DSR). Joinpoint analysis was used to examine temporal trends in the incidence of NMSCs and estimate annual percent changes (APCs). Results: During the period of 2003–2017, the incidence of NMSCs reached a direct standardized rate (DSR) of 162.62 per 100,000 in men (mortality 1.57 per 100,000) and 89.36 per 100,000 in women (mortality 0.52 per 100,000), respectively. The incidence significantly increased among both men and women across the entire period. Basal cell carcinoma (BCC), with its different morphologies, represented about 67.6% of the NMSCs in men (*n* = 2139 out of a total of 3161 tumors observed between 2013 and 2017) and about 75.8% of the NMSCs in women (*n* = 1718 out of a total of 2264 tumors from 2013 to 2017), thus accounting for the vast majority of NMSCs. The results are consistent with the literature data carried out both at national and international level. Conclusions: Proper monitoring of this phenomenon through timely reporting and recording of all new NMSC cases is necessary to develop new preventive strategies.

## 1. Introduction

The incidence of skin cancers is generally increasing worldwide due to chronic exposure to sunlight, climate change, and individual or social attitudes [1,2]. Overall, skin cancers include cutaneous melanoma (CM) and non-melanoma skin cancers (NMSC), which are mainly represented by basal cell carcinoma (BCC) and squamous cell carcinoma or spinal cell carcinoma (SCC), together with a number of rare conditions, including actinic keratoses (AK), keratoacanthoma (a squamo-proliferative tumor of uncertain malignant potential), carcinoma in situ or Bowen’s disease, and others [3]. 

NMSCs are generally considered curable diseases, but currently represent a global health problem due to their increasing incidence [4,5,6,7]. In general, non-melanoma skin cancers (NMSCs) are the most common malignancies in the world, with BCC and SCC representing over 99% of skin cancers other than melanoma [8,9]. This has led to increased attention worldwide and the development of various preventive programs. They are also known as keratinocytic skin carcinomas because they originate from keratinocytes, the main cells that build up in the epidermis. 

Specifically, skin tumors are classified according to the cells from which they originate: melanomas originate from melanocytes, while non-melanoma skin cancers (NMSCs) derive from the neoplastic transformation of keratinocytes. Basal cell carcinomas (BCCs) usually originate in sun-damaged skin from the cells of the deepest skin layers, while squamous cell carcinomas (SCCs) originate from the squamous cells of the epidermis, due to their malignant transformation. Actinic keratoses (AK) are precancerous changes to keratinocytes (involving basal keratinocytes) as a consequence of chronic sun exposure in subjects with a light skin phototype, presenting a significant risk of progression to squamous cell carcinoma (SCC). The estimated progression rate ranges from <1% to about 10%, but more recent epidemiological data indicate that more than 10% of lesions may progress to invasive SCC within 10 years and that about 60% of SCCs develop from clinically diagnosed actinic keratoses [8]. In addition, patients who have undergone organ transplantations have been shown to present a 250-fold greater relative risk of developing AK compared to immunocompetent subjects [10]. 

Squamous cell carcinoma (SCC) occurs on photodamaged skin, de novo or in association with actinic keratosis. It is a very aggressive malignant tumor that invades the dermis and can lead—in the advanced stages—to the development of metastases. About 15–35 cases per 100,000 inhabitants per year are reported worldwide, but a 2–4% annual increase is estimated as a consequence of the aging of the general population and chronic exposure to ultraviolet rays [11]. Usually, SCC originates in skin areas chronically exposed to the sun, but it can frequently develop in the lips and oral mucosa of smokers and alcohol drinkers. The risk of metastasis for lesions of the trunk and limbs varies from 2% to 5%, while for the lesions affecting the face (especially the lips, ears, tongue) and the genital region (vulva, penis), it can reach as high as 20%. SCCs with a diameter greater than 2 cm are the most aggressive. 

Keratoacanthoma (or keratoacanthoma-like squamous proliferation, as defined by the WHO) is a well-differentiated variant of SCC, usually located on the face and neck; it is of uncertain malignant potential and gives rise to a solid, isolated protuberance with a characteristic appearance. The neoplasm originates from a hair follicle or a pilosebaceous gland, develops within 6 weeks and then disappears within a few months. Carcinoma in situ (or Bowen’s disease) is a very early form of SCC in situ of the skin and typically affects genital mucous membranes (erythroplasia of Queyrat), which originates from the uncontrolled proliferation of a squamous cell, being characterized by the presence of erythematous lesions and a squamous crusted surface, often with central erosion. Basal cell carcinoma (BCC) is the most common malignant tumor of epithelial origin in the Caucasian population, and it is generally observed on the face and scalp. It is characterized by morphological polymorphism, with a high capacity of invading tissues adjacent to the site of origin. Invasiveness (with different degrees) is a characteristic common to all forms of BCC. 

This tumor does not start with premonitory lesions and develops on apparently healthy skin; it has an insidious onset, often with few specific morphological characteristics that do not allow an immediate clinical diagnosis. Natural and artificial UV rays represent the most important risk factor for the onset of BCC. The risk of systemic metastases is low, but the “morpheiform” or “scleroderma” BCC, a rarer form of this tumor (2–6%), has a high risk of recurrence due to a more pronounced infiltrative capacity. 

Morpheiform BCC is diagnosed very late due to its very slow course and poor clinical symptoms. It looks very similar to a scar, which over the years progressively increases in diameter on the skin level and can evolve into an ulcerative form or even a terebrant form. Its frequent recurrence, despite the enlarged surgical excision, underlines the aggressiveness of this variant.

Epidemiological data concerning Europe show an annual incidence rate of NMSCs is 129.3 cases in men and 90.8 in women per 100,000 inhabitants [12]. The annual incidence of BCC in Italy is approximately 100 cases per 100,000 inhabitants and represents 15% of all diagnosed cancers [13]. The Salento area in Southern Apulia could display a high impact of these kind of tumors due to the higher sun exposure of the general population for geographical and climatic reasons, as well as because of the high number of workers exposed to the sun due to their occupational duties in the sectors of summer tourism, agriculture, fisheries and building. Based on this epidemiological background, this study was aimed at computing the incidence of NMSCs and related mortality in the Province of Lecce (Southern Apulia, Italy) by examining the comprehensive real data, over a period of fifteen years (from 2003 to 2017), collected by the local cancer registry, which covers about 833,000 inhabitants potentially exposed to UV sun radiation for most of the year.

## 2. Methods

Following the STROBE guidelines, we carried out a retrospective study based on the official cancer registry compliant with international standards over a 15-year period from 2003 to 2017, in order to update the incidence of NMSCs in the Province of Lecce (Southern Apulia, Italy), with a focus on the latest 5 years (2013–2017) concerning the different histological morphologies of these tumors. The cancer patients were extracted in an anonymous form from the consultation of consolidated real-world data coming from the cancer registry implemented by the Local Health Authority ASL Lecce, compliant with the required national standard. 

The official cancer registry managed by the Local Health Authority ASL Lecce (which is a public governmental body with responsibility for healthcare services for about 800,000 people living in the entire Province of Lecce) is formally certified by the Italian Association of Cancer Registries (AIRTUM) as being compliant with the IARC standard procedures, including the appropriateness and completeness of registration. A specific session of the Registry is devoted to melanoma and non-melanoma skin cancers, based on confirmed histological diagnosis as ensured for all the other kinds of tumors. 

However, it must be disclosed that the Registry could possibly be affected by minor underreported bias due to a small proportion of skin cancer patients living in the Province of Lecce but diagnosed and treated in other Italian regions. At the same time, due to the limits of the Registry, mortality for skin cancers cannot be separately specified per type (BCC vs. SCC).

We referred to the International Classification of Oncological Diseases (ICD-0.3) third edition, which codes neoplasms according to location (topography) and histological type (morphology). The C44 code (ICD-03 805-811) specific for non-melanoma skin cancers was used, including the morphologies of invasive and non-invasive non melanoma skin tumors.

### Statistical Analyses 

The incidence for NMSC was computed as age-adjusted direct standardized rates (DSR) per 100,000 inhabitants, with 95% confidence intervals (95% CI). For DSR, we used as standard the European population for 2013 [14]. 

For the period of 2013–2017, the absolute frequencies of new NMSC annual cases were computed per each morphological type of neoplasm (Table 1, Table 2 and Table 3). 

The incidence data were also evaluated by means of descriptive analyses for the period of 2013–2017, according to mean age, median age and cumulative risk. 

The temporal trend analysis of annual standardized incidence rates was conducted through log-linear regression models, separately for men and women, by using the Joinpoint Regression Program (5.0 Version, available from: http://surveillance.cancer.gov/joinpoint, accessed on 25 May 2023), developed by the Statistical Research and Applications Branch of the United States National Cancer Institute and widely used for the analysis of trends [15]. 

To describe trends in the incidence, the annual percentage changes (APCs) in rates were estimated, with a significance level of 0.05 and 95% confidence intervals.

## 3. Results

In Table 1, we separately present the annual incidence for men and women (DSR per 100,000 inhabitants) observed across the entire study period (95% CI).

**Table 1:** DSR per 100,000 inhabitants (with CI 95%) describing the incidence of NMSCs in men and women living in the Province of Lecce (2003–2017).

	Men			Women		
Year	DSR	95% CI		DSR	95% CI	
2003	125.4	112.4	138.5	65.1	57.3	72.9
2004	143.1	129.4	156.8	62.4	54.9	69.9
2005	139.8	126.0	153.6	73.9	65.7	82.1
2006	111.4	99.7	123.1	67.4	59.7	75.1
2007	142.3	128.9	155.7	67.0	59.4	74.5
2008	137.2	124.2	150.1	64.1	56.8	71.5
2009	152.1	138.9	165.3	77.9	69.9	86.0
2010	148.1	135.3	161.0	80.1	72.0	88.1
2011	146.7	134.1	159.3	76.9	69.0	84.8
2012	140.1	128.0	152.2	71.2	63.7	78.7
2013	169.8	156.5	183.0	83.1	75.0	91.2
2014	167.7	154.8	180.6	90.7	82.3	99.1
2015	160.5	147.9	173.1	87.0	78.8	95.2
2016	156.8	144.4	169.1	95.0	86.5	103.6
2017	159.3	147.0	171.6	90.8	82.5	99.2

**Figure 1:**
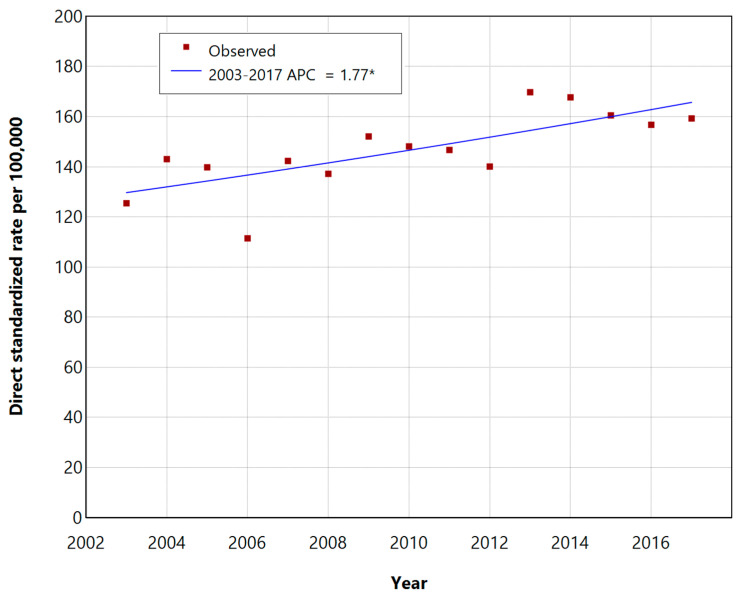
Incidence trend of NMSCs in men of the Province of Lecce during the period of 2003–2017, based on the DSR per 100,000 inhabitants, presented with annual percentage changes (APCs) * Indicates that the APC is significantly different from zero at the 0.05 level.

**Figure 2:**
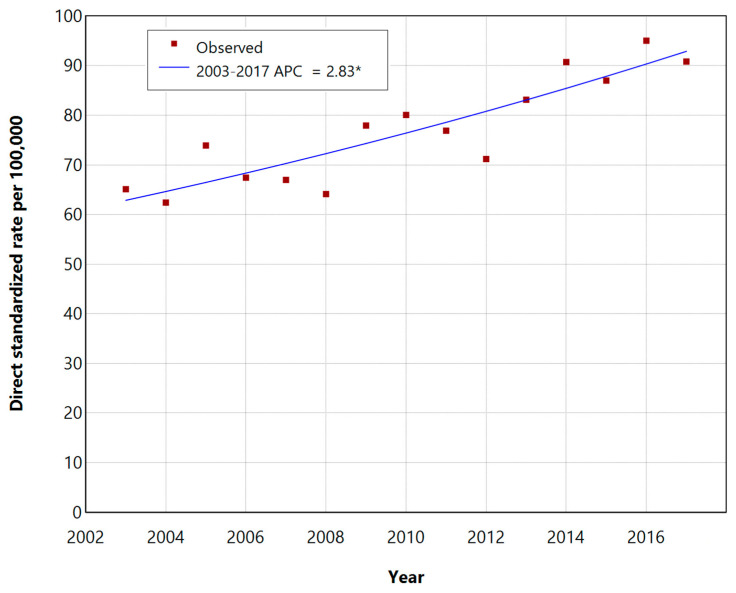
Incidence trend of NMSCs in women of the Province of Lecce during the period of 2003–2017, based on the DSR per 100,000 inhabitants, presented with annual percentage change (APC). * Indicates that the APC is significantly different from zero at the 0.05 level.

The trends in the incidence of DSR during the period of 2003–2017 with the annual percentage changes (APCs) are shown in Figure 1 and Figure 2, respectively, for men and women.

The standardized incidence rates of NMSCs increased over the 15-year period in both groups, showing statistically significant APCs of 1.77% (95% CI: 0.74–3.01, *p*-value < 0.05) for males and 2.83% for women (95% CI: 1.82–3.98, *p*-value < 0.05), respectively.

Based on histologically confirmed diagnoses reported by the Cancer Registry of Local Health Authority of Lecce, we recorded a total of 5425 NMSC cases in the 5 years from 2013 to 2017 (3161 in men and 2264 in women) among the total population of the Province of Lecce of about 830,000 inhabitants. 

The total number and percentages of NSMC cases per each morphological type of neoplasm are presented in Table 2 (men), Table 3 (women) and Table 4 (overall males + females). Basal cell carcinoma (BCC), with its different morphologies, represented about 67.6% of the NMSCs in men and about 75.8% of the NMSCs in women, thus accounting for the vast majority of NMSCs. 

It should be noted that metatypical and basosquamous skin cancers are considered the same by some authors, meaning that the WHO has started to recommend including both under the definition of basosquamous tumors.

**Table 2:** Non-melanoma skin cancer in the Province of Lecce in the years 2013–2017. Total cases recorded in men and frequency of microscopically confirmed morphologies.

	Diagnosis	N	%
1	80903 Basal Cell Carcinoma (not specified)	1735	54.89
2	80703 Squamous Cell Carcinoma	523	16.55
3	80973 Nodular Basal Cell Carcinoma	232	7.34
4	80923 Invasive Basal Cell Carcinoma	172	5.44
5	80943 Basal-Squamous Cell Carcinoma	127	4.02
6	80913 Superficial Basal Cell Carcinoma	86	2.72
7	80953 Metatypic Carcinoma	101	3.2
8	80713 Keratinic Squamous Cell Carcinoma	70	2.21
9	80763 Micro-invasive Squamous Cell Carcinoma	32	1.01
10	Other Morphologies	83	2.63
	**Total NMSCs in Men (2013–2017)**	3161	100

**Table 3:** Non-melanoma skin cancer in the Province of Lecce in the years 2013–2017. Total cases recorded in women and frequency of microscopically confirmed morphologies.

	Diagnosis	N	%
1	80903 Basal Cell Carcinoma (not specified)	1349	59.58
2	80703 Squamous Cell Carcinoma	290	12.81
3	80973 Nodular Basal Cell Carcinoma	164	7.24
4	80923 Invasive Basal Cell Carcinoma	112	4.95
5	80943 Basal-Squamous Cell Carcinoma	72	3.18
6	80913 Superficial Basal Cell Carcinoma	93	4.11
7	80953 Metatypic Carcinoma	63	2.78
8	80713 Keratinic Squamous Cell Carcinoma	37	1.63
9	80763 Micro-invasive Squamous Cell Carcinoma	16	0.71
10	Other Morphologies	68	3
	**Total NMSCs in Women (2013–2017)**	2264	100

**Table 4:** Non-melanoma skin cancer in the Province of Lecce in the years 2013–2017. Total cases recorded in men + women and frequency of microscopically confirmed morphologies.

	Diagnosis	N	%
1	80903 Basal Cell Carcinoma (not specified)	3084	56.85
2	80703 Squamous Cell Carcinoma	813	14.99
3	80973 Nodular Basal Cell Carcinoma	396	7.3
4	80923 Invasive Basal Cell Carcinoma	284	5.24
5	80943 Basal-Squamous Cell Carcinoma	199	3.67
6	80913 Superficial Basal Cell Carcinoma	179	3.3
7	80953 Metatypic Carcinoma	164	3.02
8	80713 Keratinic Squamous Cell Carcinoma	107	1.97
9	80763 Micro-invasive Squamous Cell Carcinoma	48	0.88
10	Other Morphologies	151	2.78
	**Total NMSCs in Men + Women (2013–2017)**	5425	100

Among patients who had received a diagnosis of NMSC, there were 30 deaths in men and 16 in women, for a total of 46 patients in the entire 5-year period (2013–2017). The data reported show a higher incidence and mortality associated with NMSC in men than in women, with the latter showing higher annual growth. There were no significant differences concerning mean and median age between men and women. A total of 40 cases (29 in females and 11 in males) were recorded in younger people aged between 15 and 29 years old.

## 4. Discussion

NMSCs’ incidence is increasing worldwide and represents the most relevant current dermatological oncology problem, together with cutaneous melanoma. The purpose of the present research was to update incidence and mortality data relating to NMSCs in the Province of Lecce (Southern Apulia, Italy) by using real-world data coming from the Local Cancer Registry of Lecce and its province, covering a total of 831,000 inhabitants. The specific objective was to compare the data with the data present in the literature referring to the Italian and European situation, with the aim of directing the attention of researchers and professionals in this field and reporting of these tumors, highlighting the importance of early diagnosis to obtain satisfactory results in terms of minimally invasive therapeutic possibilities and prognosis. In the frame of further prospective studies, it would be interesting to study the appearance of new events in the study population, such as complications and possible recurrences, and to analyze the genetic, environmental and personal risk factors [16] that can influence this trend. It would be useful to deepen the study of this phenomenon by dividing the population under examination into age groups and evaluating the annual increasing rate for each one. In our study, mortality data were presented overall, as aggregated information (mortality for all types of NMSC) without assessing the specific mortality of BCC and SCC separately, due to the intrinsic limitations of our cancer registry in providing detailed mortality data per cancer type. However, it is known that mortality is almost or entirely confined to SCC. An increase in the incidence rate of NMSCs has been reported globally, being associated with several factors, including the transition to significantly older populations, higher risk of NMSC, and increased exposure to sunlight [17,18,19]. Research has revealed the important role of increased occupational and recreational exposure to UV light in the pathogenesis of NMSCs [20,21]. Women younger than 40 years old showed a consistent increase in BCC incidence rates of 6.3% between 1973 and 2009 [22]. Other studies have shown that indoor tanning is associated with a significantly higher risk of BCC and SCC, with an even higher risk if artificial exposure occurred before 25 years old [23].

The mechanisms leading to the development of NMSCs are multifactorial and exposure to UV rays represents the most important risk factor, with a latency period of 15–20 years between exposure to UV rays and the onset of the disease [18]. Chronic or intermittent exposure to UV radiation, especially in fair-skinned individuals, is known to trigger approximately 90% of NMSCs, causing malignant transformation of keratinocytes and the suppression of the inflammatory response. However, several complex genotypic, phenotypic, and environmental factors contribute to the pathogenesis of these cancers. Sunburn, especially in childhood, immunosuppressive conditions, and the use of antihypertensive drugs (e.g., hydrochlorthiazide) or cholesterol-lowering drugs have been proved to have a carcinogenic action. Other risk factors for the development of BCC and SCC include concomitant diseases and treatments (e.g., psoriasis), chronic exposure to human papillomavirus, drug-induced immune suppression in patients undergoing organ transplantation, targeted agents for the treatment of other cancers [9,10,19], systemic immunosuppression, chemotherapy, and radiation therapy [24]. UVRs, in particular, are able to determine DNA damage and the development of somatic mutations, inflammation, oxidative stress and altered activity of cells of the immune system. These events are crucial for the development of skin cancers.

However, UVAs and UVBs induce different skin alterations: UVAs promote deeper damage to the skin and destroy DNA indirectly, causing oxidative stress with the formation of free radicals; UVBs cause erythema, directly damaging the DNA, with the formation of thymine dimers, cyclobutane pyrimidine dimers (CPD) and 6–4 photopro-ducts [25,26,27,28]. The most important mutation is that affecting the p53 tumor suppressor, causing its inactivation and therefore the inability of the cell to undergo programmed cell death, with the consequent uncontrolled cell growth responsible for the tumor [29].

The growing incidence and morbidity of skin cancers other than melanoma has generated great interest among researchers. Understanding the pathogenetic mechanisms underlying the development of these tumors is of fundamental importance for the development of new and more effective treatments. While the role of cumulative sun exposure in the pathogenesis of squamous cell carcinoma appears clear, the relationship between sun exposure patterns and various subtypes of basal cell carcinoma remains undetermined. Also, unlike basal cell carcinoma, squamous cell carcinomas can result from precursor lesions. The diagnosis of non-melanoma skin cancer is made clinically and confirmed by histological testing. 

The prognosis depends on the characteristics of the lesion and on the physiological and immune status of the host. These conditions also influence the choice of treatment. Another issue is represented by the skin neoplasms subsequent to the first one, as many people with a clear phototype present more than five NMSCs over a 10-year period. Surgical excision with predetermined margins is the mainstay of treatment for squamous cell carcinoma and most basal cell carcinomas. Other treatment modalities include physical destruction (radiotherapy, curettage and curettage, and cryotherapy), chemical destruction (photodynamic therapy and topical 5-flurouracil), and immunomodulatory therapy (topical imiquimod). Among new non-invasive treatments, only photodynamic therapy and topical imiquimod have become established treatments for specific subtypes of basal cell carcinoma. The aim of the research is to consolidate new medical therapies that are less invasive and more tissue-conserving [10,11]. Preventive strategies are aimed at reducing sun exposure, but are of unproven benefit, especially for basal cell carcinoma.

In Italy, the low aggressiveness of most of these tumors, combined with the diagnosis being temporally distant from the onset and often being carried out in an outpatient setting (outside the public hospital system), have led in many situations to an underestimation of the number of cases, with strong fluctuations in the rates of incidence by region. For these reasons, skin cancers are not usually included in the calculation of incidence, survival and prevalence indicators periodically published by cancer registries, and there are no unified national data. Despite the increase in their incidence, NMSCs are characterized by a relatively low mortality rate. 

Compared to malignant melanoma, which has a mortality rate of 2.3% within the first year from the diagnosis, they are characterized by a lower mortality rate but a considerably higher incidence [28].

Primary prevention programs mainly involve the development and promotion of adequate photoprotection and photo-exposure strategies. The use of protective creams containing sunscreens represents one, but not the only, method of protection, with the use of protective clothing, hats and glasses, as well as avoiding exposure to direct sunlight while spending part of the day in the shade, also being recommended. Particular attention must be paid to subjects particularly at risk, i.e., children and people with a light phototype. The same measures also make it possible to prevent the onset of other skin cancers, such as melanomas [30,31,32,33].

The key tool of secondary prevention, on the other hand, is screening. A screening test is a test that allows for the early detection of a certain disease, specifically a tumor, in asymptomatic people. In some cases, screening can prevent the onset of cancer, and in others it can save their life. When this is not possible, early diagnosis increases therapeutic opportunities and—in any case—allows minimally invasive and non-destructive interventions to be carried out, reducing the negative effects of the disease [30,31,32,33]. The activation of population screening programs is supported both nationally and internationally. The term “oncological screening” indicates the availability of specific tests to detect the onset of tumors early. These tests are conducted on apparently healthy people—that is, those who have no signs or symptoms of cancer. Free screening programs are currently active in Italy for three types of cancer: cervical, breast and colorectal. As regards the secondary prevention of NMSCs, however, there are currently no real screening programs on a national scale. However, particularly in patients with risk factors such as a light phototype, advanced age, chronic exposure to the sun and immunosuppression, a dermatological visit every 6–12 months can allow early recognition and treatment. The dermatological visit, in addition to the clinical examination, should include a dermoscopic examination to increase diagnostic sensitivity and specificity, i.e., reduce the risk of not diagnosing malignant lesions or treating benign lesions [21].

## 5. Conclusions

Cancer registries represent a valuable and reliable tool to determine the real incidence of neoplasms in a defined population, as they analyze the epidemiology of a specific tumor in order to develop proper health policies. Information provided by the Cancer Registry of the Local Health Authority of Lecce allowed us to update the incidence of NMSCs and related mortality in this province (covering about 800,000 people), whose population is assumed to experience heavy and frequent sun exposure due to climatic/environmental factors, both during work and leisure. Proper monitoring of this phenomenon through timely reporting and recording of all new NMSC cases is necessary to develop new preventive strategies.

## Data Availability

Data, in the form of anonymous statistics can be made available upon request via email to uose@asl.lecce.it.
